# Covalent Grafting of the RGD-Peptide onto Polyetheretherketone Surfaces via Schiff Base Formation

**DOI:** 10.1155/2013/616535

**Published:** 2013-10-21

**Authors:** Marc Becker, Steffen Lorenz, Dennis Strand, Christian-Friedrich Vahl, Matthias Gabriel

**Affiliations:** ^1^I. Medical Department, University Medical Center of the Johannes Gutenberg University, 55131 Mainz, Germany; ^2^FZI, I. Med and Policlinic, Johannes Gutenberg University School of Medicine, Obere Zahlbacher Street 63, 55131 Mainz, Germany; ^3^Department of Cardiothoracic and Vascular Surgery, Johannes Gutenberg University School of Medicine, Langenbeckstraße 1, 55131 Mainz, Germany

## Abstract

In recent years, the synthetic polymer polyetheretherketone (PEEK) has increasingly been used in a number of orthopedic implementations, due to its excellent mechanical properties, bioinertness, and chemical resistance. For *in vivo* applications, the surface of PEEK, which does not naturally support cell adhesion, has to be modified to improve tissue integration. In the present work we demonstrate a novel wet-chemical modification of PEEK to modify the surface, enabling the covalent grafting of the cell-adhesive RGD-peptide. Modification of the polymer surface was achieved via Schiff base formation using an aliphatic diamine and subsequent crosslinker-mediated immobilization of the peptide. In cell culture experiments with primary osteoblasts it was shown that the RGD-modified PEEK not only significantly promoted cellular adhesion but also strongly enhanced the proliferation of osteoblasts on the modified polymer surface.

## 1. Introduction

High performance polymers increasingly find their use in applications which have until now been the realm of metallic materials. Notably the polymer polyetheretherketone (PEEK) shows great promise as an alternative for titanium and its alloys in the therapy of skeletal dysfunctions in orthopedics and trauma surgery [1]. The characteristics of PEEK include a high melting temperature (approx. 335°C) and a high glass transition temperature (approx. 135°C), far above the body temperature, which means that creeping is not an issue. The mechanical properties match more closely that of bone compared to titanium. In addition PEEK exhibits high chemical resistance and low abrasiveness and is considered to be bioinert [1]. PEEK—in contrast to metallic materials—is radiolucent which facilitates X-ray investigation. However, the poor ability of the polymer to support cellular adhesion is unfavorable for many applications where tissue integration is desired. In order to alter this property, numerous attempts have been performed to endow PEEK surfaces with appropriate qualities. Plasma technology was used to enhance the attachment of fibroblasts and bone cells to modified material [2]. Basic research using various photochemical routes was explored which may open up further ways for the enhancement of cell adhesiveness [3]. A highly promising and purely wet-chemical approach is based upon the generation of hydroxy functionalities via reduction of the keto groups of the benzophenone repeating units of the PEEK polymer backbone in order to generate hydroxy functions [4]. This method served as a base for the covalent immobilization of the highly cell-adhesive protein fibronectin.

The motivation for the present work was to provide a facile technique for the wet-chemical modification of PEEK, with a view to enhance biocompatibility. This was achieved by the formation of a Schiff base using an aliphatic diamine and the subsequent conjugation of the cell-adhesive RGD-peptide onto the activated surface.

## 2. Material and Methods

Polyether ether ketone (PEEK) was purchased from Technoplast (Lahnstein, Germany) as 12 mm round stock material. Standard chemicals, ethylene diamine (EDA), diethylene glycol diglycidyl ether (DGE), nuclear stain Hoechst 33342, and the peptide Arg-Gly-Asp-Ser (RGD) were obtained from Sigma-Aldrich (Steinheim, Germany). Sulfosuccinimidyl-4-o-(4,4-dimethoxytrityl) butyrate (sulfo-SDTB) was provided by VWR (Darmstadt, Germany). Wet abrasive paper was purchased from usual commercial sources except 4000 grit paper which was obtained from Patho-Service (Oststeinbek, Germany). Cy-5 NHS-ester was provided by Amersham (München, Germany). Aqueous solutions were prepared with 18 M*Ω* water. Plastic ware was purchased from Greiner Bio-One (Frickenhausen, Germany).

## 3. Preparation, Surface Activation, and RGD Grafting of PEEK

Samples of 3 mm thickness were cut from PEEK rods and ground with wet abrasive paper in ascending order (400, 800, 1200, 2500, and 4000 grit). Discs were cleaned with water and degreased with isopropanol. Samples were immersed in neat EDA and treated for various periods (1, 2, 3, and 4 h) at 120°C, followed by thorough washing with water and isopropanol. Static water contact angles of aminated samples were determined on a Krüss DSA 10-MK2 (Krüss Optronic, Hamburg, Germany). Amino groups on aminated samples were quantified with the colorimetric sulfo-SDTB assay according to the manufacturer's instructions [5]. The equipment used for the determination of surface roughness of bare and EDA-treated samples was a KLA Tencor P 16+ (Milpitas, CA, USA) with a scan rate of 50 *μ*m/s at 100 Hz, a needle weight of 1 mg, and a tip diameter of about 0.1 *μ*m. The scan length was about 500 *μ*m. Attenuated Total Reflection Fourier Transform Infrared (ATR-FTIR) spectra were obtained on a Nicolet Magna-IR 850 (Nicolet, Dreieich, Germany). Immobilization of the RGD-peptide was achieved by first incubating amine-bearing specimen in 5% (v/v) DGE in 50 mM carbonate buffer (pH 9) for 2 h and subsequent washing with water. Grafting of the peptide was accomplished by reacting this surface with 0.2 mg/mL RGD in carbonate buffer overnight [6]. Cleaned samples were sterilized in 60% iso-propanol for 30 min prior to use in cell culture experiments.

## 4. Cell Culture

Primary human osteoblasts were isolated from the femoral head of 3 healthy donors undergoing total hip arthroplasty. Informed consent was obtained from all patients and from the local human ethical committee. Bone fragments were washed at least 5 times with PBS and digested with collagenase type IV (Sigma-Aldrich, Steinheim, Germany) for 30 min at 37°C. After digestion bone fragments were washed 5 times with PBS and placed in six-well plates with DMEM/F12 + GlutaMAX (Gibco, Life Technologies, Carlsbad, CA USA), supplemented with 10% heat inactivated FCS (PAA Lab, Pasching, Austria), penicillin (100 U/mL), streptomycin (100 *μ*g/mL), dexamethasone (10^−8^ M), ascorbate (50 *μ*g/mL), and *β*-glycerophosphate (3.5 mM). Medium was changed twice a week. Cells from passage 2–4 were seeded on both untreated and RGD-modified samples at a concentration of 5 × 10^4^ cells in 2 mL medium. Cy5-NHS and Hoechst 33342 stock solutions were prepared with dimethylsulfoxide (dried over 3 Å molecular sieve) at 1 mg/mL. Samples were washed with sterile PBS and incubated in 2 mL PBS containing 2 *μ*L of each dye stock solution for 2 h at 37°C. Stained specimens were again washed with PBS and immediately examined under a Zeiss LSM 710 confocal microscope (Zeiss, Oberkochen, Germany). Excitation/emission wavelengths were 405/450 nm for Hoechst 33342 and 633/680 nm for the Cy5-dye.

## 5. Results

**Figure 1:**
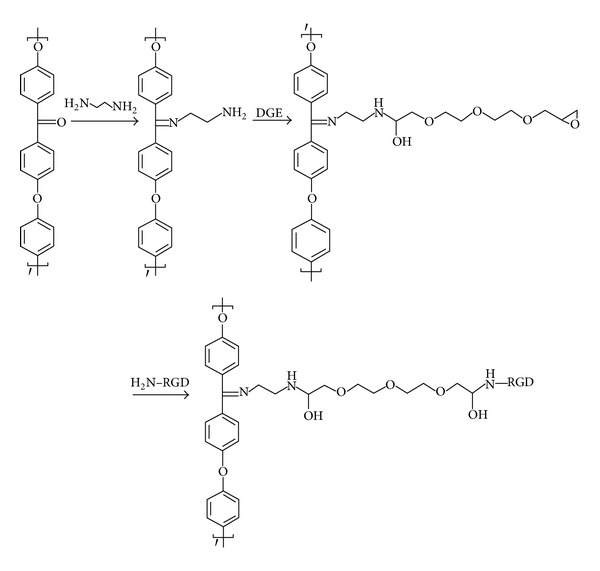
Reaction scheme for the cell-adhesive modification of PEEK. Surface activation was achieved via Schiff base formation using a diamine. Subsequent coupling of a diepoxide crosslinker afforded the covalent conjugation of the RGD-peptide.

**Figure 2:**
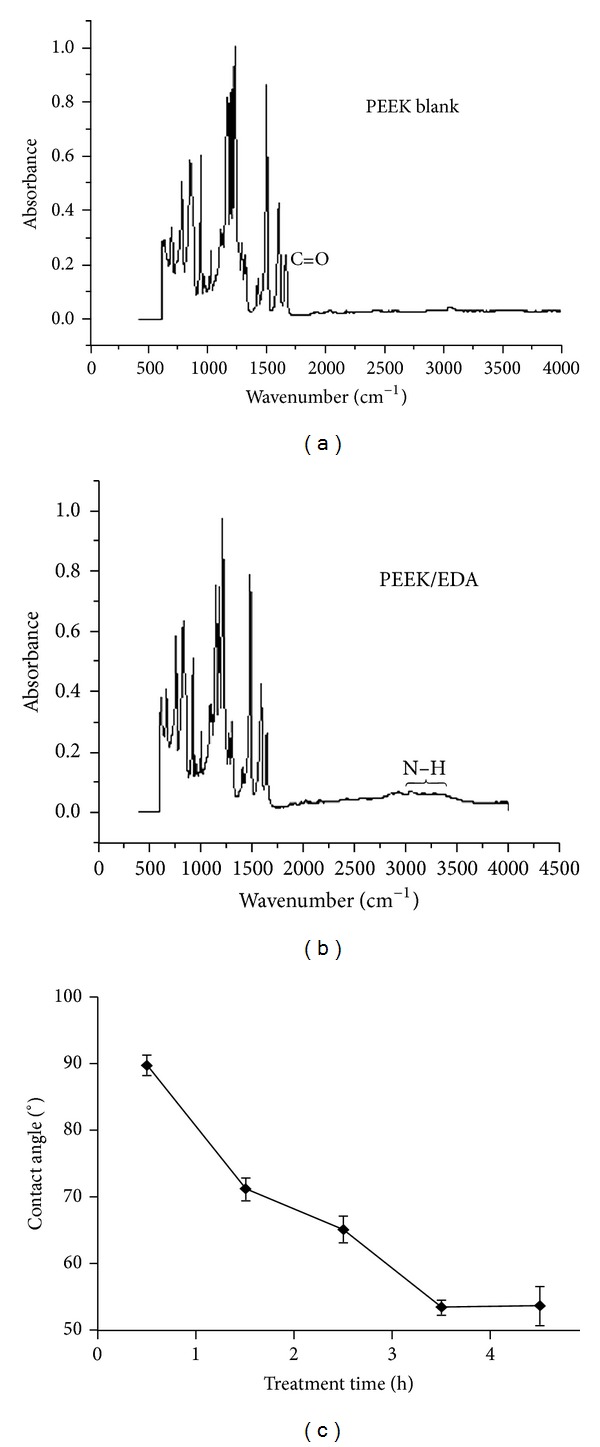
ATR-FTIR and contact angle measurement. As a consequence of the diamine treatment a signal emerged around 3400 cm^−1^ suggesting the formation of amino moieties (b) that is not present on unmodified material (a). Due to extensive hydrogen bonding this peak is extremely broadened. Wettability increases in the course of treatment time (c) and reaches a maximum after 3 h indicating a maximum of surface modification.

The formation of a Schiff base accompanied by the generation of pendant primary amino groups was used to activate PEEK surfaces as depicted in Figure 1. The time course of the reaction was monitored by contact angle measurements (Figure 2(c)). A maximum modification was achieved after 3 h incubation under the chosen conditions indicated by a minimum of the contact angle. Quantification of surface amines via the colorimetric sulfo-SDTB assay yielded 3.6 ± 1.27 NH_2_/nm^2^ from triplicate determinations.

Surface roughness (root mean square) of untreated samples was 28 ± 4.93 nm and 32 ± 5.68 nm, respectively, for EDA-activated material. 

Infrared spectroscopy revealed a pronounced signal at 3400 cm^−1^ emerging as a result of the amination reaction (Figures 2(a) and 2(b)). This signal, indicating N–H bonds, was extremely broadened due to hydrogen bonding. Further interpretation—for example, of the fingerprint region—was not feasible.

**Figure 3:**
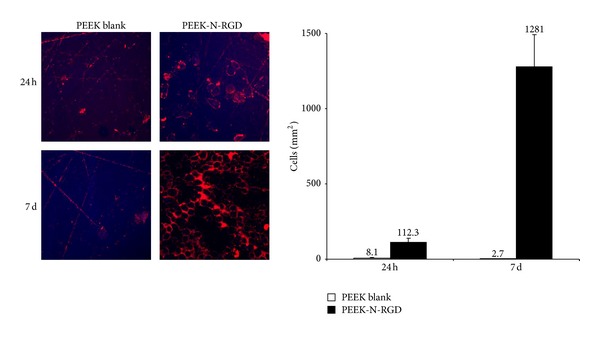
Adhesion and growth of primary human osteoblasts on bare and RGD-modified PEEK surfaces. Cells were cultured for 24 h or 7 d. After cultivation, cell coverage was analyzed by fluorescence microscopy. Results from triplicate determinations are expressed as mean ± SD (magnification ×100, bar = 20 *μ*m).

Cell culture on bare and RGD-modified polymer was performed for the initial attachment (24 h) phase and for a 1-week period. The adhered osteoblasts were visualized indirectly by staining with the long-wavelength fluorescent dye Cy5. Staining with Hoechst 33342 did not show reproducible results (see discussion “autofluorescence”).  Figure 3 shows cellular adhesion as a function of the surface chemistry. While untreated PEEK showed almost negligible colonization by osteoblasts even after 1 week, the RGD-immobilized samples exhibited excellent support for cellular adhesion (112 ± 26.5 cells/mm^2^ after 24 h) and proliferation (1281 ± 210 cells/mm^2^ after 1 week).

## 6. Discussion

Among various approaches for surface modifications of PEEK, the wet-chemical methods may be considered as more generally applicable. In contrast to photochemical and plasma technologies which suffer from limitations regarding the treatment of geometrically demanding structures (pores, tubing, undercut structures, etc.) liquid based modifications are mostly feasible. Noiset et al. utilized chemical reduction with a hydride to convert the keto functions of the polymer into secondary alcohols which were subsequently reacted with a diisocyanate serving as a spacer molecule [4]. Free isocyanate moieties then served to conjugate amine-containing molecules. In this work, the formation of a Schiff base (ketamine) between the keto groups of PEEK and one amino portion of ethylenediamine was meant to serve the same purpose. There is comparatively sparse literature on the formation of ketamine of PEEK, and—to our knowledge—this approach has not yet been used to surface-activate the polymer for biomedical applications [7, 8]. In general this reaction requires elevated temperatures possibly due to steric hindrance of the benzophenone moiety of the polymer backbone which on the other hand prevents the hydrolysis of the ketamine in an aqueous environment, making reductive amination unnecessary. Optimization of the treatment time was monitored by contact angle measurements assuming that maximum wettability is accompanied by maximum density of the polar amino functions. We previously used this method for the modification of a different polymer and yielded a comparable density of amino functionalities [6]. Within the accuracy of the measurement the surface topology was not altered by the chemical treatment. The subsequent conjugation of the hydrophilic spacer DGE and the ultimate coupling of the RGD-peptide were performed in an aqueous buffer. Overall, the entire procedure is relatively fast and less prone to the presence of moisture compared to the reduction-diisocyanate procedure. Bare PEEK does not support cellular attachment whereas RGD-modified polymer surfaces showed excellent adhesion promoting properties. We used the minimal adhesive motif RGD found in many proteins (e.g., collagen type I and fibronectin) of the extracellular matrix for the modification because of the ease of handling of a small peptide—especially concerning sterilization—in comparison to a whole protein. PEEK exhibits an extraordinarily strong autofluorescence (not shown) which prohibited the use of standard green or red fluorescent dyes for cell staining. To circumvent this limitation we used a labeling procedure with an amine-reactive derivative of the long-wavelength dye Cy5. By this method the cells are not labeled directly but membrane proteins and secreted components were stained, yielding a negative picture of the cellular distribution. As a possible substitute for titanium, PEEK matches more closely the mechanical properties of bone. In order to achieve an improved osseointegration the RGD-grafted surface was tested with bone cells for initial attachment (24 h) and proliferation (7 d). These results, obtained *in vitro*, may be further elucidated in animal experiments under realistic *in vivo* conditions.

## 7. Conclusion

The increasing utilization of PEEK for example, as a substitute for titanium, requires methods to endow the polymer with cell-adhesive properties. A wet-chemical technique was developed that is based upon a ketamine-based activation of the PEEK surface in combination with a crosslinker-mediated conjugation of the RGD-peptide. The presented method, which is feasible for the laboratory scale, may serve as a promising application for an enhanced performance of medical devices, fabricated from PEEK.
